# A124 INITIAL EXPERIENCES AND OUTCOMES IN LAUNCHING AN ESD PROGRAM IN CANADA

**DOI:** 10.1093/jcag/gwab049.123

**Published:** 2022-02-21

**Authors:** T Jeyalingam, N Forbes, S Heitman, P J Belletrutti

**Affiliations:** Division of Gastroenterology and Hepatology, University of Calgary, Calgary, AB, Canada

## Abstract

**Background:**

Endoscopic submucosal dissection (ESD) is emerging as the preferred modality for curative resection of certain gastrointestinal lesions, yet it has a steep learning curve. Formal ESD training in Canada is limited, thus several endoscopists have sought training abroad. There are no data on the initial outcomes of ESD in Canada.

**Aims:**

To describe the experience of a single advanced endoscopist (P.B.) who completed a mentor-apprentice model of ESD training in Europe and recently launched an ESD service in Canada.

**Methods:**

Formal training in submucosal endoscopy was completed over a 12-month period (Sep 2018-Aug 2019) in Milan, Italy involving a stepwise progression through case observation (n=40), use of animal models (n=10), partial completion of human cases under direct supervision (n=25), and independent completion of human cases (n=19). Upon return to Calgary, AB, the program was promoted via grand rounds, an evening launch event and word-of-mouth. Patient and lesion characteristics, as well as procedure details and ESD outcomes were recorded prospectively in a database and used to complete a practice audit. Cases were divided into two timeframes (first 18 vs second 18 cases) and then compared with respect to specimen size, resection speed, R0 resection rate, and adverse event (AE) rate using the Mann-Whitney U and two-proportion Z-tests.

**Results:**

From Nov 2019 to Sep 2021, 36 lesions were treated by ESD in 34 patients (mean (SD) age=66.5 (13.5) years). Most lesions were located in the rectum (44.4%) or stomach (41.7%); 21 (58.3%) were neoplastic, of which 5 (23.8%) were submucosal carcinomas, 11 (52.3%) were mucosal carcinomas (7 m1/Tis, 4 m2-3/T1a), and 5 (23.8%) were neuroendocrine tumors. The overall en bloc resection rate was 97.2% (35/36 lesions). In comparing the first 18 cases to the second 18 cases (Table 1), there were non-significant trends toward increases in mean specimen size (5.4 to 8.6 cm^2^, P=0.07) and R0 resection rate (62.5% to 84.6%, P=0.24). Resection speed increased significantly (2.97 to 6.25 cm^2^/hr, P=0.04), while AE rates remained the same (11.1%); namely 2 episodes of bleeding requiring repeat endoscopy in each set of cases. There were no perforations or intra-abdominal infections.

**Conclusions:**

With appropriate training and support, ESD can be performed safely and effectively in a Canadian context. Proficiency can be expected to improve significantly within the first 2 years of independent ESD practice without compromising safety.

**Funding Agencies:**

None

**Table:** Table 1.

ALL LOCATIONS	1st 18 cases	2nd 18 cases	All 36 cases	P
Elliptical area (cm2)	5.35	8.59	7.74	0.07
Total ESD time (min)	94.28	97.72	96.00	0.70
Resection speed (cm2/hr)	2.97	6.25	4.61	0.04*
R0 resection (%)	62.50	84.62	76.19	0.24
Adverse events (%)	11.11	11.11	11.11	1

